# The treatment choices of abdominal aortic aneurysm patients in China in the era of value-based healthcare

**DOI:** 10.3389/fcvm.2022.961830

**Published:** 2022-11-29

**Authors:** Liren Duan, Wei Xin, Shenli Li, Lin Zhao, Shijie Xin

**Affiliations:** ^1^Department of Vascular Surgery, The First Affiliated Hospital of China Medical University, Shenyang, China; ^2^Key Laboratory of Pathogenesis, Prevention and Therapeutics of Aortic Aneurysm, Shenyang, China; ^3^Department of Operation Management, The First Affiliated Hospital of China Medical University, Shenyang, China; ^4^Department of Anesthesiology, The People's Hospital of Liaoning Province, Shenyang, China; ^5^Department of Pharmacology, China Medical University, Shenyang, China

**Keywords:** abdominal aortic aneurysm (AAA), value-based healthcare delivery, open repair, endovascular AAA repair, China

## Abstract

**Background:**

Endovascular aneurysm repair (EVAR) is often seen as the first choice treatment for patients with abdominal aortic aneurysm (AAA), particularly high-risk patients, yet the long-term survival rate and improvement in quality of life are still unclear. In order to seek the value of EVAR to the entire healthcare field, we conducted a retrospective study to evaluate whether the improvement EVAR can truly bring to the quality of medical care in the era of value-based healthcare.

**Methods:**

We included AAA patients who underwent surgical treatment in the Department of Vascular Surgery, First Hospital of China Medical University, from January 1, 2004, to December 31, 2019 and evaluated surgery procedure data, short-term and long-term mortality, complications, prognoses, and medical costs.

**Results:**

We analyzed 507 patients with AAA who underwent open repair (*n* = 232) or EVAR (*n* = 275) over a 15-year period. The operative time, blood loss, blood transfusion rate, and postoperative length of hospital stay of the EVAR group is significantly lower than which of the open repair group. Meanwhile, neither short-term nor long-term mortality rates shows significant differences between the two groups. On the other hand, the complication rate of the EVAR group was significantly higher than that of the open repair group. Lastly, the total cost of EVAR was significantly higher than that of open repair.

**Conclusion:**

Existing evidence suggests that EVAR improves neither short-term nor long-term survival rate compared with open surgery. In contrast, the complication rate and the reintervention rate in the EVAR group were higher than those in the open surgery group. Moreover, the cost of EVAR and that paid by medical insurance were higher than those for open surgery. For patients with a long-life expectancy, in order to ensure that patients receive appropriate and effective care, surgeons should choose a suitable method that considers both the quality of medical care as well as the expense accordingly.

## Background

Abdominal aortic aneurysm (AAA) is a localized, permanent, abnormal expansion of the abdominal aorta. When the diameter of the dilated abdominal aorta exceeds 1.5 times its normal diameter, AAA is diagnosed. Continuous dilation and total rupture are the usual final outcomes of AAA, and once an AAA ruptures, the mortality rate is 80–90% (1–6). According to the guidelines of relevant vascular surgery associations, when the maximum diameter of the AAA exceeds 5.5 cm, surgical repair is usually necessary. To treat AAA there are currently two surgical options. One is open repair, which was first performed in in 1951 by Dubost, and the other is endovascular aneurysm repair (EVAR), which was first performed in 1991 by Parodi. EVAR is associated with faster recovery and is less invasive than open repair, so most vascular centers have adopted EVAR as their first choice method for AAA repair.

Many studies have shown that EVAR has an early survival advantage compared with open repair; however, long-term outcomes still requires further evaluation. A meta-analysis of four randomized trials, namely, the EVAR-1, DREAM, OVER and ACE trails, indicated that 3 years after aneurysm repair, aneurysm-related mortality was five times higher in the EVAR group than in the open surgery group, likely contributing to the increased mortality in the EVAR group in the long term (7–11). In the EVAR-2 trail, indicated that EVAR did not improve survival over no intervention to the patients already unfit for open repair (12). In such case, with the popularization of EVAR in China, reviewing the management of Chinese patients with AAA is necessary.

The epidemiological AAA data of Asian populations and Caucasian populations are quite different (13–17). Relevant data show that the average age of male patients with AAA in central China is 57 years old, which is far younger than the average age of AAA patients in developed countries (18). According to the World Health Organization (WHO) report, Chinese citizens have an average life expectancy of 76 years, which means that the average expected survival time of Chinese AAA patients after EVAR is nearly 20 years. The durability of a stent graft is 10 years according to the Food and Drug Administration (FDA) recommendation, which means that for patients aged <65 years, the rates of stent-related complications and subsequent reinterventions incremented over time, leading to an increased treatment cost (19). The data presented in our previous report showed that the treatment cost of EVAR was significantly higher than that of open surgery (20). China's public health expenditure is growing rapidly every year, therefore balancing medical costs and the quality of medical care is a challenge for current medical reforms. To date, most articles have compared clinical data associated with the surgical repair of AAA; however, selecting the surgical method that costs the least with the most benefits is still an important decision concerning both doctors and patients. In this article, we comprehensively consider patients' postoperative quality of life and public health economics in order to discuss the surgical decision-making process for Chinese patients with AAA.

## Methods

### Patients

This study is a retrospective study of surgical treatment in the Department of Vascular Surgery, the First Hospital of China Medical University, conducted from January 1, 2004, to December 31, 2019. The study was approved by the Ethics Committee of the First Hospital of China Medical University, and written informed consent was obtained from all patients. The conditions for the inclusion of patients were applicable to both EVAR and open repair. All the patients received the infrarenal fixation, and the type of endograft in the EVAR group was Endurant (Medtronic). The choice of surgical method was left to the patient's discretion. The Society for Vascular Surgery (SVS) guideline was used to define aneurysm: an AAA with a diameter ≥5.5 cm on computed tomography (CT). Patients who are suitable for only one surgical method were excluded from this study. A total of 507 patients were included in this study. We prospectively and systematically collected basic data, surgical information, and hospitalization-related information of the patients and recorded comorbidities, secondary interventions during follow-up, and death.

### Preoperative and postoperative data

The case notes of all patients were retrieved. Data relating to demographics and comorbidities were extracted. Operative data included total operation time, blood loss, blood transfusion, and length of total postoperative stay. All patients who underwent EVAR underwent contrast or non-contrast CT during follow-up. And the patients treated with EVAR were in instruction for use for the specific endograft. The deadline for follow-up was December 31, 2021. All patients were followed up for at least 2 years. All radiological images were reviewed by an independent radiologist for evidence of endoleaks, deformation, graft-related infection, thrombosis, graft fracture, and graft migration, and diameter measurements were obtained. All patients who underwent open repair were followed with serial clinical examinations and imaging with color duplex scanning, CT, or magnetic resonance angiography (MRA).

### Statistical analysis

Descriptive statistics were calculated as means, standard deviations, ranges, and proportions as appropriate. χ^2^ or Fisher's exact tests were used to compare nominal variables between the two groups as appropriate.

Logistic regression models were used to analyze the association between the type of surgical procedure and 30-day complication outcomes. Multiple-variable logistic regression models were constructed to adjust for age and other risk factors, including sex. The univariate and multivariate odds ratios (EVAR vs. open repair) with 95% confidence intervals (CIs) are reported for the surgical repair variable.

Primary patency, primary assisted patency, secondary patency, reintervention, and freedom from a combined failure endpoint incorporating AAA-related death, AAA rupture, and conversion were estimated with the Kaplan-Meier survival method. Significance tests comparing the two groups were performed with log-rank tests. Multiple-variable Cox proportional hazards models were used to adjust for possible confounding factors in the analyses of primary patency and reintervention. The univariate and multivariate hazard ratios (EVAR vs. open repair) and the associated 95% CIs were reported for the type of surgical repair. A *P*-value of < 0.05 was considered statistically significant in all analyses.

## Preoperative and postoperative results of the two surgical methods

### Patient baseline data

During the 15-year period, 232 patients underwent open repair, and 275 patients underwent EVAR for AAA. The number of EVARs increased, whereas the number of open repairs decreased over time. In terms of demographics (Table 1), the groups were evenly matched in terms of sex. The average age of the open repair group was 61.7 ± 9.4 years, and that of the EVAR group was 69.0 ± 8.8 years. Males comprised over 80% of the total population. There were no significant differences in the variables of hypertension, dyslipidemia, diabetes mellitus, smoking, history of myocardial infarction, or peripheral vascular disease between the two groups. EVAR application for AAA repair increased as patient age increases, and open repair application decreased as age increases. In the 60–65 age group, the proportions of EVAR and open repair were nearly equal. We divided them into two groups accordingly, the younger group is consisted of patients with age 65 or younger and the elder group includes patients of age older than 65. The baseline data of the two groups are shown in Tables 2, 3.

**Table 1:** Demographics and comorbidities of patients undergoing open repair or EVAR for AAA during 2004–2019.

**Variables**	**Open repair**	**EVAR**
	**(*n* = 232)**	**(*n* = 275)**
Male	189 (81.5%)	220 (80.0%)
Female	43 (18.5%)	55 (20.0%)
Age	61.7 ± 9.4	69.0 ± 8.8
Hypertension	141 (60.8%)	162 (58.9%)
Dyslipidemia	93 (40.1%)	107 (38.9%)
Diabetes mellitus	38 (16.4%)	42 (15.3%)
Smoking	138 (59.5%)	166 (60.4%)
History of myocardial infarction	45 (19.4%)	62 (22.5%)
Peripheral vascular disease	32 (13.8%)	43 (15.6%)

Continuous data are presented as means ± standard deviations; categorical data are presented as counts (percentages).

**Table 2:** Demographics and comorbidities of patients undergoing open repair or EVAR for AAA during 2004–2019 in young and middle-aged group.

**Variables**	**Open repair**	**EVAR**
	**(*n* = 139)**	**(*n* = 79)**
Male	108 (77.7%)	65 (82.3%)
Female	31 (22.3%)	14 (17.7%)
Age	55.7 ± 6.4	58.1 ± 5.5
Hypertension	91 (65.5%)	42 (53.2%)
Dyslipidemia	39 (28.1%)	17 (21.5%)
Diabetes mellitus	32 (23.0%)	16 (20.3%)
Smoking	66 (47.5%)	35 (44.3%)
History of myocardial infarction	26 (18.7%)	15 (19.0%)
Peripheral vascular disease	14 (10.1%)	7 (8.9%)

Continuous data are presented as means ± standard deviations; categorical data are presented as counts (percentages).

**Table 3:** Demographics and comorbidities of patients undergoing open repair or EVAR for AAA during 2004–2019 in elder group.

**Variables**	**Open repair**	**EVAR**
	**(*n* = 93)**	**(*n* = 196)**
Male	81 (87.1%)	155 (79.1%)
Female	12 (12.9%)	41 (20.9%)
Age	70.7 ± 4.9	73.3 ± 9.4
Hypertension	50 (53.8%)	120 (61.2%)
Dyslipidemia	54 (58.1%)	90 (45.9%)
Diabetes mellitus	6 (6.5%)	26 (13.3)
Smoking	72 (77.4%)	131 (66.8%)
History of myocardial infarction	19 (20.4%)	47 (24.0%)
Peripheral vascular disease	18 (19.4%)	36 (18.4%)

Continuous data are presented as means ± standard deviations; categorical data are presented as counts (percentages).

### Analysis of results after open surgery and EVAR

#### Perioperative outcomes

Many foreign studies have reported the advantages of EVAR during the perioperative period. The results from our center indicated that compared with open surgery, EVAR was associated with a significantly shorter operative time, less intraoperative blood loss, a lower incidence of intraoperative blood transfusion, and a shorter postoperative hospital length of stay (Table 4). A meta-analysis of our previous report summarized the efficacy of AAA repair in China (21) (Table 5) and indicated that EVAR also had obvious advantages in the perioperative period. Another review that included 40 domestically relevant studies also supported this result (22). Thus, Chinese clinical data indicate that the intraoperative trauma associated with EVAR during the perioperative period is significantly less than that of open surgery, and the recovery of patients after EVAR is significantly faster than that of patients after open surgery.

**Table 4:** Perioperative outcomes.

**Variables**	**Open repair**	**EVAR**
	**(*n* = 232)**	**(*n* = 275)**
Operative time (min)	210	156
Blood loss (ml)	583	79
Blood transfusion (ml)	416	0
Length of total postoperative stay (days)	16	11

**Table 5:** Perioperative outcomes.

**Variables**	**Open repair**	**EVAR**
	**(*n* = 1,757)**	**(*n* = 1,105)**
Operative time (min)	261.9	183.8
Blood loss (ml)	965.3	156.8
Blood transfusion (ml)	694.5	52.3
ICU stay (hours)	64.3	31.1
Length of total postoperative stay (days)	14.9	11

#### Postoperative long-term follow-up results

##### Analysis of postoperative survival

**Figure 1:**
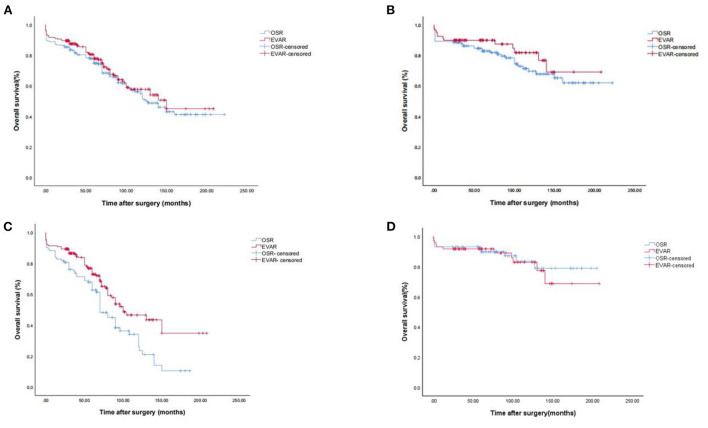
Kaplan-Meier survival analysis for AAA. **(A)** Kaplan-Meier survival analysis for AAA. **(B)** Kaplan-Meier survival analysis for AAA in young and middle-aged group. **(C)** Kaplan-Meier survival analysis for AAA in elder group. **(D)** Kaplan-Meier survival analysis for AAA in young and middle-aged group (PSM analysis).

There was no statistically significant difference in mortality during hospitalization between the two surgical groups (*P* > 0.05; Table 6). According to the Kaplan-Meier curve (Figure 1A), the median survival time in the open repair group was 125 months, and that in the EVAR group was 150 months. We then applied a Kaplan Meier survival analysis to different age groups (Figures 1B,C). Table 6 lists the causes of in-hospital death and death after discharge. In the adjusted analyses using Cox proportional hazard models, the major factors related to survival were age, dyslipidemia, smoking and a history of myocardial infarction. Type of surgery, sex, hypertension, diabetes mellitus, and peripheral vascular disease were not related to survival (Table 7). Moreover, we then applied Propensity Score Matching (PSM) analysis to avoid the age factor in young and middle-aged group, and the results showed that there were no significant differences in survival rate (Figure 1D).

**Table 6:** Postoperative long-term follow-up results.

	**Open repair**	**EVAR**
Any death	95	81
In-hospital death	24	18
Aneurysm-related	15/24 (62.5%)	10/18 (55.6%)
Cardiovascular, non-aneurysm-related	5/24 (20.8%)	5/18 (27.8%)
Other	4/24 (16.7%)	3/18 (16.7%)
After discharge	71	63
Aneurysm-related rupture	0/71 (0%)	8/63 (12.7%)
Aneurysm-related, non-rupture	1/71 (1.4%)	5/63 (7.9%)
Cardiovascular, non-aneurysm	25/71 (35.2%)	22/63 (34.9%)
Cancer	35/71 (49.3%)	20/63 (31.7%)
Other	10/71 (14.1%)	8/63 (12.7%)

**Table 7:** Hazard ratios (HRs) and proportional hazards model (multivariate model) results for the overall survival rate of AAA surgery.

**Covariate**	**HR**	**95% CI**	***P*** **-value**
EVAR (vs. open repair)	1.355	0.965–1.902	0.079
Sex	0.879	0.591–1.309	0.527
Age	1.041	1.019–1.062	< 0.01
Hypertension	1.226	0.864–1.740	0.253
Dyslipidemia	4.406	2.635–7.367	< 0.01
Diabetes mellitus	0.681	0.395–1.173	0.166
Smoking	7.153	3.169–16.146	< 0.01
History of myocardial infarction	2.214	1.603–3.057	< 0.01
Peripheral vascular disease	0.910	0.618–1.338	0.631

##### Analysis of reintervention after EVAR

During the follow-up period, among the 275 EVAR patients, 28 reinterventions were performed in 24 (8.7%) patients. The numbers and causes of reinterventions are summarized in Table 8. In the cohort, 8.0% patients required 1 reintervention, and 0.7% patients required multiple reinterventions. Endoleaks (Type II), deformation, infection and thrombosis were responsible for 25.0% (7), 21.4% (6), 14.3% (4), and 7.1% (3) of the total reinterventions, respectively; fracture, migration and aneurysm rupture accounted for 10.7% (3) of the reinterventions. There is currently no secondary intervention for open surgery in our center due to related complications.

**Table 8:** Reintervention rate in the EVAR group after discharge due to graft-related complications.

**Reasons**	**No. (percentage)**
Endoleak	7 (25.0%)
Deformation	6 (21.4%)
Infection	4 (14.3%)
Aneurysm rupture	3 (10.7%)
Fracture	3 (10.7%)
Migration	3 (10.7%)
Thrombosis	2 (7.1%)
Total	28

##### Single-center health economics analysis

Due to factors such as inflation and exchange rate fluctuation, in this analysis, we calculated only the medical prices and medical insurance reimbursement prices for our center for the past 5 years. Medical expenditures across the country are roughly the same; therefore, the data from our center can approximately reflect the national situation. In our health economic analysis (Table 9), the total cost of EVAR was significantly higher than that of the open surgery (EVAR: an average of US$33,915; open surgery: an average of US$15,937). The amounts of medical insurance reimbursement were US$10,662 and US$8,974 in the EVAR and open repair groups, respectively; in terms of out-of-pocket expenses, patients who underwent EVAR paid US$23,289, and patients who underwent open surgery paid US$6,963 dollars. We analyzed the cost of the graft (stent graft/artificial vessel), and the results showed that the cost of the stent graft in the EVAR group was US$17,502, and the cost of the artificial vessel in the open repair group was US$2,831. We further calculated the cost of surgery; the average cost of EVAR was US$1,843, and the average cost of open surgery was US$1,574. The average cost of drug treatment was US$1,757 in the EVAR group and US$4,085 in the open repair group. The average cost of examination was US$853 in the EVAR group and US$1,155 in the open repair group. Due to large differences in the hospital lengths of stay in each group of patients admitted to the ICU, the total costs were significantly different. Therefore, we calculated the daily cost of the two surgical methods for ICU patients. The average daily cost of EVAR in ICU patients was US$1,120, and the average daily cost of open surgery was US$2,103. In terms of postoperative follow-up, the average cost of each follow-up for EVAR patients $307, and the average cost of each follow-up for open surgery patients was $86.

**Table 9:** The costs of EVAR and open repair.

**Cost (USD)**	**EVAR**	**Open repair**
Total cost	33,951	15,937
Insurance coverage	10,662	8,974
Out-of-pocket amount	23,289	6,963
Stent graft cost (artificial vessel)	17,502	2,831
Surgery cost	1,843	1,574
Drug cost	1,757	4,085
Examination cost	853	1,155
ICU (per day)	1,120	2,103
Follow-up cost (per event)	307	86

## Discussion

Data from our center and relevant domestic literature indicate that trauma associated with EVAR in the perioperative period is significantly less than that of open surgery. However, the survival rates in the perioperative and long-term follow-up periods were not significantly different between the EVAR and open surgery groups. The medical cost of EVAR is significantly higher than that of open surgery, and the secondary intervention rate after EVAR is also significantly higher than that after open surgery. This result suggests that in China, the benefits of EVAR are consistent with those in the United States and other countries; however, whether it should be the first choice for the treatment of AAA or not needs to be reflected upon. Differences in epidemiology, anatomy, basic comorbidities, and socioeconomic status among different races will affect the choice of cardiovascular disease treatment options and postoperative efficacies (23–25). Epidemiological characteristics between China and the United States are quite different, and the age at onset in Chinese people is significantly lower than that in Americans (4, 18). The economic statuses of Western countries and China are also quite different. Moreover, the medical insurance policy models of the two countries are also different, and these differences possess a strong impact on the choice of surgery. In the era of value medicine, it is important to pay more attention to the choice of surgical method in order to improve the quality of medical care for AAA patients. In this article, we discuss the treatment decision-making options for AAA patients in China based on medical quality and health economics.

Due to its minimal invasiveness, EVAR is currently more frequently used in Western countries than in China. Data from our research and other domestic reports show that compared with open surgery, EVAR significantly reduces trauma to patients, especially patients with advanced age or a poor basic condition, and the advantages are obvious. However, it worthies noting in the aspect of mortality rate since our results revealed the fact that no significant difference in perioperative mortality between the EVAR and open surgery groups. This result is similar to the annual report of the Japanese SVS (26). The reason of this phenomenon may be due to the increasing trend of EVAR application in cases of hemodynamic instability, suggesting that in critically ill patients, although EVAR can reduce surgical trauma, it showed no significant effect on the survival rate during the perioperative period. We combined the long-term follow-up results from our center and the domestic data; similarly, the results showed that EVAR did not improve patients' long-term survival rates.

As the first EVAR was performed in only 1991, EVAR has been performed for <30 years, which means that for this new technique, long-term follow-up studies may not be comprehensive. According to the research results from our center and data from other clinical centers in China, the secondary intervention rate in EVAR patients is significantly higher than that in open surgery patients. Among them, endoleak is the most common complication of EVAR surgeries (27–29). The world's first EVAR patient who received the Parodi treatment required a second intervention due to a IB endoleak. In addition to endoleaks, stent displacement, deformation and graft thrombosis are also common complications. Our vascular surgery center has performed some repeated interventions, and these complications after EVAR not only result in pain due to reintervention but also increased medical expenses due to repeated surgeries.

The reintervention rate in EVAR patients increased with postoperative time is valueless. Therefore, for patients with younger age and a longer life expectancy, the incidence of complications related to EVAR surgery and the secondary intervention rate may be increased. According to FDA requirements, the fatigue and durability of the current stent graft is 10 years. A 20-year follow-up study of patients with first-generation stents reported that more than 80% of patients required secondary intervention treatment, although the current stent manufacturing process is constantly changing. To date, the complication rate associated with stents is lower than it was previously, but the material is still the same as that used in the first generation; the main component is nitinol (30). Our long-term follow-up results showed that after AAA repair, ~90% of patients died of conditions not related to AAA. This suggests that AAA had no significant impact on the life expectancy of patients. The report by Sweeting et al. (31) also confirmed that the survival time in younger patients is also often longer than that in older patients. This means that for young and middle-aged patients, more frequent follow-ups and reintervention may be required after 10 years after EVAR. The long-term quality of life decreases, and medical costs increase.

In recent years, medical expenses in various countries have continued to rise, which has led to an increase in public health and individual medical expenditures; therefore, how to control the rapid and unreasonable increase in health economic expenditures has become a challenge that governments need to solve. In this article, we analyzed open surgery and EVAR health economics data from Shenyang, a city in north-eastern China, as an example to estimate the general situation of the country. According to our results, patients who received EVAR had to pay an average of US$16,326 more than those who received an open surgery. According to incomplete statistics derived by Chinese scholars, there are more than 5,000 EVARs performed in China each year, while fewer than 500 open repair surgeries are carried out in China. The proportion of EVARs carried out in China is close to 90%. We also calculated the proportion of EVARs in other countries. Table 10 shows the difference between the country with the lowest EVAR rate (Hungary, 28%) and the country with the highest EVAR rate the (United States, 79%). Further analysis found that this may be related to the medical insurance reimbursement model (32). Countries based on fees for services, such as the United States and Germany, perform more EVARs than countries with population-based reimbursement programs, such as Hungary. This shows that socioeconomic status and different medical insurance models have significant impacts on the choice of medical methods. Judging from the current data, the utilization rate of EVAR in China exceeds that of most countries in the world. Considering China's current economic level and medical insurance reimbursement model, we still need to consider the decision-making process regarding AAA patients' surgical methods.

**Table 10:** National EVAR proportions of AAA repair.

**Country (district)**	**Proportion of EVAR**	**Mean age (years)**
United States	79.5%	72.8
Germany	77%	74.0
Korea	73.9%	63.5
Australia	73.7%	74.6
Sweden	56.8%	72.4
Iceland	53.9%	72.6
New Zealand	51.7%	73.8
Finland	46.2%	72.2
Denmark	33.9%	71.6
Norway	32.0%	71.4
Hungary	27.8%	68.9

The increasing use of EVAR also reflects the profound medical problems associated with surgeon training. Compared with that of open surgery, the difficulty of EVAR is much less, and the learning curve time is significantly reduced, which means that a surgeon can master EVAR in a short time. Accordingly, vascular surgeons, interventionists and cardiologists can perform EVAR, and these doctors always perform only EVAR, leading to the large-scale application of this type of operation. According to the guidelines, the application of EVAR has strict indications. The guidelines of the National Institute of Health and Clinical Optimization (NICE) in the United Kingdom note that when treating complex AAAs, open surgery instead of EVAR is recommended. However, in clinical practice, we often perform off-label application of EVAR for different indications due to many factors, which also leads to the abuse of EVAR. Therefore, in terms of vascular surgeon training, we should train vascular surgeons to perform open surgery as well as EVAR.

When new therapies and medical technologies are applied in clinical work, there is often a phase of early unrealistic optimism, followed by the identification of downsides. Our results suggest that in young and middle-aged patients, the advantage associated with the minimal invasiveness of EVAR is not obvious. In contrast, the high incidence of postoperative complications requires frequent follow-up, and the risk of reintervention, which also reduces the quality of medical care and increases medical expenses, is contradictory to the concept of value-based healthcare. In line with the current economic level and medical insurance policy in China, we believe that when making surgical decisions for young and middle-aged AAA patients, we should re-examine the options and consider open surgery, which can not only save medical expenses but also reduce the risk of secondary intervention after surgery, potentially significantly improving the quality of life of patients. Despite the limitations of the above studies, we believe that the results are still of great significance. We look forward to performing large-scale studies with strict standards and additional joint clinical trials to improve the validity of the research results.

## Conclusion

The goal of value-based healthcare is to obtain the maximum medical value with the least medical cost. It had been reported that 20–40% of medical resources worldwide are wasted. Relevant research shows that advanced technology and increased costs have not resulted in improved medical quality; this is known as the so-called medical diminishing marginal effect. Our research shows that in young and middle-aged patients, compared with traditional open repair surgery, EVAR was not associated with obvious advantages; in contrast, it reduced the quality of life of patients in the long term. Therefore, our research results suggest that in the future, after comprehensive consideration, we should consider open surgery instead of EVAR for young and middle-aged patients.

## Data availability statement

The original contributions presented in the study are included in the article/supplementary material, further inquiries can be directed to the corresponding author.

## Ethics statement

The studies involving human participants were reviewed and approved by the First Hospital of China Medical University. The patients/participants provided their written informed consent to participate in this study.

## Author contributions

LD, SL, and SX contributed to conception and design of the study. WX organized the database. LZ performed the statistical analysis. LD wrote the first draft of the manuscript. SL, WX, LZ, and SX wrote sections of the manuscript. All authors contributed to manuscript revision, read, and approved the submitted version.

## Funding

This study was supported by grants from the National Natural Science Foundation of China (Nos. 81974049 and 81770488).

## Conflict of interest

The authors declare that the research was conducted in the absence of any commercial or financial relationships that could be construed as a potential conflict of interest.

## Publisher's note

All claims expressed in this article are solely those of the authors and do not necessarily represent those of their affiliated organizations, or those of the publisher, the editors and the reviewers. Any product that may be evaluated in this article, or claim that may be made by its manufacturer, is not guaranteed or endorsed by the publisher.

## References

[1] Thompson SG Ashton HA Gao L Buxton MJ Scott RA. Final follow-up of the Multicentre Aneurysm Screening Study (MASS) randomized trial of abdominal aortic aneurysm screening. Br J Surg. (2012) 99:1649–56. 10.1002/bjs.8897 23034729 PMC3569614

[2] Lindholt JS Juul S Fasting H Henneberg EW. Preliminary ten year results from a randomised single centre mass screening trial for abdominal aortic aneurysm. Eur J Vasc Endovasc Surg. (2006) 32:608–14. 10.1016/j.ejvs.2006.06.008 16893663

[3] Kuivaniemi H Elmore JR. Opportunities in abdominal aortic aneurysm research: epidemiology, genetics, and pathophysiology. Ann Vasc Surg. (2012) 26:862–70. 10.1016/j.avsg.2012.02.005 22794334

[4] Kent KC Zwolak RM Egorova NN Riles TS Manganaro A Moskowitz AJ. Analysis of risk factors for abdominal aortic aneurysm in a cohort of more than 3 million individuals. J Vasc Surg. (2010) 52:539–48. 10.1016/j.jvs.2010.05.090 20630687

[5] Al-Balah A Goodall R Salciccioli JD Marshall DC Shalhoub J. Mortality from abdominal aortic aneurysm: trends in European Union 15+ countries from 1990 to 2017. Br J Surg. (2020) 2020:11635. 10.1002/bjs.11635 32391589

[6] Baxter BT Terrin MC Dalman RL. Medical management of small abdominal aortic aneurysms. Circulation. (2008) 117:1883–9. 10.1161/CIRCULATIONAHA.107.735274 18391122 PMC4148043

[7] Greenhalgh RM. Comparison of endovascular aneurysm repair with open repair in patients with abdominal aortic aneurysm (EVAR trial 1), 30-day operative mortality results: randomised controlled trial. Lancet. (2004) 364:843–48. 10.1016/S0140-6736(04)16979-1 15351191

[8] Blankensteijn JD de Jong SE Prinssen M van der Ham AC Buth J van Sterkenburg SM. Two-year outcomes after conventional or endovascular repair of abdominal aortic aneurysms. N Engl J Med. (2005) 352:2398–405. 10.1056/NEJMoa051255 15944424

[9] Lederle FA Freischlag JA Kyriakides TC Matsumura JS Padberg FT Jr Kohler TR. Long-term comparison of endovascular and open repair of abdominal aortic aneurysm. N Engl J Med. (2012) 367:1988–97. 10.1056/NEJMoa1207481 23171095

[10] Becquemin JP Pillet JC Lescalie F Sapoval M Goueffic Y Lermusiaux P. A randomized controlled trial of endovascular aneurysm repair versus open surgery for abdominal aortic aneurysms in low- to moderate-risk patients. J Vasc Surg. (2011) 53:1167–73.e1. 10.1016/j.jvs.2010.10.124 21276681

[11] Powell JT Sweeting MJ Ulug P Blankensteijn JD Lederle FA Becquemin JP. Meta-analysis of individual-patient data from EVAR-1, DREAM, OVER and ACE trials comparing outcomes of endovascular or open repair for abdominal aortic aneurysm over 5 years. Br J Surg. (2017) 104:166–78. 10.1002/bjs.10430 28160528 PMC5299468

[12] EVAR trial participants. Endovascular aneurysm repair and outcome in patients unfit for open repair of abdominal aortic aneurysm (EVAR trial 2): randomised controlled trial. Lancet. (2005) 365:2187–92. 10.1016/S0140-6736(05)66628-7 15978926

[13] Yii MK. Epidemiology of abdominal aortic aneurysm in an Asian population. ANZ J Surg. (2003) 73:393–5. 10.1046/j.1445-2197.2003.t01-1-02657.x 12801335

[14] Poon JT Cheng SW Wong JS Ting AC. Prevalence of abdominal aortic aneurysm in Chinese patients with severe coronary artery disease. ANZ J Surg. (2010) 80:630–3. 10.1111/j.1445-2197.2010.05345.x 20840407

[15] Adachi K Iwasawa T Ono T. Screening for abdominal aortic aneurysms during a basic medical checkup in residents of a Japanese rural community. Surg Today. (2000) 30:594–9. 10.1007/s005950070098 10930224

[16] Salem MK Rayt HS Hussey G Rafelt S Nelson CP Sayers RD. Should Asian men be included in abdominal aortic aneurysm screening programmes? Eur J Vasc Endovasc Surg. (2009) 38:748–9. 10.1016/j.ejvs.2009.07.012 19666232

[17] Benson RA Poole R Murray S Moxey P Loftus IM. Screening results from a large United Kingdom abdominal aortic aneurysm screening center in the context of optimizing United Kingdom National Abdominal Aortic Aneurysm Screening Programme protocols. J Vasc Surg. (2016) 63:301–4. 10.1016/j.jvs.2015.08.091 26482996

[18] Li K Zhang K Li T Zhai S. Primary results of abdominal aortic aneurysm screening in the at-risk residents in middle China. BMC Cardiovasc Disord. (2018) 18:60. 10.1186/s12872-018-0793-5 29614976 PMC5883536

[19] Choi K Han Y Ko GY Cho YP Kwon TW. Early and late outcomes of endovascular aortic aneurysm repair versus open surgical repair of an abdominal aortic aneurysm: a single-center study. Ann Vasc Surg. (2018) 51:187–91. 10.1016/j.avsg.2018.02.042 29772312

[20] Yue WL Xin SJ Zhang J Xiao L Hu HD Hu XH. The study of different operative style for abdominal aortic aneurysm during surrounding operation. Zhonghua Yi Xue Za Zhi. (2010) 90:1309–12. 10.3760/cma.j.issn.0376-2491.2010.19.005 20646577

[21] Shi F He Y Wang S Jia F Ji C Zhang J. Endovascular and open surgical repair of abdominal aortic aneurysms: a comparative analysis of western and Chinese studies. Rev Cardiovasc Med. (2020) 21:75–92. 10.31083/j.rcm.2020.01.513 32259906

[22] Wang SW Lin Y Yao C Lin PL Wang SM. Comparison of clinical curative effect between open surgery and endovascular repair of abdominal aortic aneurysm in China. Chin Med J. (2012) 125:1824–31. 10.3760/cma.j.issn.0366-6999.2012.10.025 22800907

[23] Osborne NH Upchurch GR Jr Mathur AK Dimick JB. Explaining racial disparities in mortality after abdominal aortic aneurysm repair. J Vasc Surg. (2009) 50:709–13. 10.1016/j.jvs.2009.05.020 19703760

[24] Epstein AJ Gray BH Schlesinger M. Racial and ethnic differences in the use of high-volume hospitals and surgeons. Arch Surg. (2010) 145:179–86. 10.1001/archsurg.2009.268 20157087

[25] Wilson CT Fisher E Welch HG. Racial disparities in abdominal aortic aneurysm repair among male medicare beneficiaries. Arch Surg. (2008) 143:506–10. 10.1001/archsurg.143.5.506 18490563

[26] The Japanese Society for Vascular Surgery Database Management Committee Member and NCD Vascular Surgery Data Analysis Team. Vascular surgery in Japan: 2013 annual report by the Japanese society for vascular surgery. Ann Vasc Dis. (2019) 12:566–86. 10.3400/avd.ar.19-00109 31942223 PMC6957902

[27] Liaw JV Clark M Gibbs R Jenkins M Cheshire N Hamady M. Update: complications and management of infrarenal EVAR. Eur J Radiol. (2009) 71:541–51. 10.1016/j.ejrad.2008.05.015 18614311

[28] Golzarian J Struyven J. Imaging of complications after endoluminal treatment of abdominal aortic aneurysms. Eur Radiol. (2001) 11:2244–51. 10.1007/s003300100912 11702167

[29] Chaikof EL Dalman RL Eskandari MK Jackson BM Lee WA Mansour MA. The society for vascular surgery practice guidelines on the care of patients with an abdominal aortic aneurysm. J Vasc Surg. (2018) 67:2–77.e2. 10.1016/j.jvs.2017.10.044 29268916

[30] Väärämäki S Salenius J Pimenoff G Uurto I Suominen V. Overall outcome after endovascular aneurysm repair with a first-generation stent graft (Vanguard): a 20-year single-center experience. J Vasc Surg. (2020) 72:896–903. 10.1016/j.jvs.2019.11.027 32139310

[31] Sweeting MJ Patel R Powell JT Greenhalgh RM. Endovascular repair of abdominal aortic aneurysm in patients physically ineligible for open repair: very long-term follow-up in the EVAR-2 randomized controlled trial. Ann Surg. (2017) 266:713–19. 10.1097/SLA.0000000000002392 28742684

[32] Beck AW Sedrakyan A Mao J Venermo M Faizer R Debus S. Variations in abdominal aortic aneurysm care: a report from the international consortium of vascular registries. Circulation. (2016) 134:1948–58. 10.1161/CIRCULATIONAHA.116.024870 27784712 PMC5147037

